# Microwave-Assisted Hierarchically Grown Flake-like NiCo Layered Double Hydroxide Nanosheets on Transitioned Polystyrene towards Triboelectricity-Driven Self-Charging Hybrid Supercapacitors

**DOI:** 10.3390/polym15020454

**Published:** 2023-01-15

**Authors:** Seungju Jo, Narasimharao Kitchamsetti, Hyunwoo Cho, Daewon Kim

**Affiliations:** 1 Department of Electronics and Information Convergence Engineering, Kyung Hee University, 1732 Deogyeong-daero, Giheung-gu, Yongin 17104, Republic of Korea; 2 Department of Electronic Engineering, Kyung Hee University, 1732 Deogyeong-daero, Giheung-gu, Yongin 17104, Republic of Korea; 3 Institute for Wearable Convergence Electronics, Kyung Hee University, 1732 Deogyeong-daero, Giheung-gu, Yongin 17104, Republic of Korea

**Keywords:** polystyrene, microwave, layered double hydroxide (LDH), energy conversion and storage

## Abstract

Recently, there is a need to explore the utilization of various heterostructures using the designed nanocomposites and tuning the surfaces of electrodes for improving the electrochemical performance of supercapacitors (SC). In this work, a novel approach is successfully employed through a facile two-step synthetic route with the assistance of a microwave for only 1 min. Depending on the glass transition of a polystyrene (PS) substrate and electrochemical deposition (ECD) of electroactive Ni-Co layered double hydroxides (LDHs), a hierarchically designed flake-like morphology can be readily prepared to enhance the surface-active sites, which allows a rhombohedral Ni-Co LDHs electrode to obtain superior electrochemical properties. Further, the interactions between electrode and electrolyte during the diffusion of ions are highly simplified using multiple enhanced electroactive sites and shorter pathways for electron transfer. The unique surface architecture of the PS substrate and the synergistic effect of the bimetallic components in Ni-Co LDHs enable this substrate to obtain desired electrochemical activity in charge storage systems. The optimized MWC Co_0.5_Ni_0.5_ electrode exhibited an areal capacity of 100 µAh/cm^2^ at a current density of 1 mA/cm^2^ and a remarkable capacity retention of 91.2% over 5000 continuous charging and discharging cycles due to its remarkable synergistic effect of abundant faradaic redox reaction kinetics. The HSC device is assembled with the combination of optimized MWC Co_0.5_Ni_0.5_ and activated carbon as a positive and negative electrode, respectively. Further, the electrochemical test results demonstrated that MWC Co_0.5_Ni_0.5_ //AC HSC device showed a high areal capacitance of 531.25 mF/cm^2^ at a current density of 5 mA/cm^2^. In addition, the fabricated an aqueous HSC device showed a power density of 16 mW/cm^2^ at an energy density of 0.058 mWh/cm^2^, along with the remarkable capacity retention of 82.8% even after 10,000 continuous charging and discharging cycles. Moreover, the assembled hybrid supercapacitor (HSC) device is integrated with a triboelectric nanogenerator (TENG) for the development of energy conversion and storage systems. Not only an extensive survey of materials but also an innovative solution for recent progress can confirm the wide range of potential SC applications. Remarkably, this study is a new way of constructing self-powered energy storage systems in the field of sustainable wearable electronics and future smart sensing systems.

## 1. Introduction

To meet the demands of sustainable development and renewable energy utilization, advanced energy storage systems have been extensively investigated and attracted considerable interest from researchers across the world. For this, supercapacitors (SCs), with their numerous advantages such as higher power density, faster charge–discharge rates, longer lifetime, and lower maintenance cost [1,2,3,4] are promising alternatives to batteries. The SCs can be categorized into two types according to the charge storage mechanism: electrical double-layer capacitors (EDLCs) and pseudo-capacitors. EDLCs can store charge based on physical charge accumulation and non-faradaic mechanisms. Due to their large surface area, good electric conductivity, and easy accessibility, carbon nanomaterials with inherent porous structures are utilized to drive highly accessible electrolyte penetration [5,6,7,8,9]. Another side, the charge storage in pseudo-capacitors depends on the fast and reversible faradaic redox reactions on the surface of electroactive materials. Mostly, metal oxides and conducting polymers of pseudocapacitive materials are suggested to achieve excellent electrical conductivity with high specific surface area [10,11,12,13]. One drawback is that it is necessary to solve the limited energy density, which stems from the relatively fast discharging time of SCs [14,15,16,17,18,19,20,21,22].

To treat these issues, various transition metal oxides/hydroxides (TMOs/TMHs) type electrodes have become promising approaches to achieve advanced electrochemical activity compared to EDLCs type electrode materials, leading to enhanced capacitance, cycle life, power, and energy density [23,24,25,26,27,28,29]. In terms of easily controllable surface morphology and chemical composition, TMOs/TMHs are studied to be the competitive materials for most representative electroactive materials. Accordingly, a great deal of effort has been put into exploring advanced strategies to accelerate the kinetics of TMO/TMH materials [30,31,32,33,34]. The development of novel nanostructures can result in a large surface area, more electroactive sites, and short ionic diffusion paths, leading to good conductivity as well as enhanced structural stability. 

It should be noted that various nanostructures of electroactive materials facilitate efficient ion/electron transport [35,36,37,38]. Through synthesis of different morphologies and nanostructures such as nanowires [39,40,41,42], nanorods [43,44,45], nanotubes [46,47], nanosheets [48], and hollow spheres [49,50,51], the ion diffusion pathways can be shortened, and the electroactive surface area increased. As a result, excellent electrochemical efficiency and high charge storage capability can be obtained via abundant electroactive sites. Recently, the development of faradaic redox materials has been studied to offer superior capacity, rich redox activity, and higher electrochemical performance. For example, N. Kitchamsetti et al. [1] reported a core–shell heterostructured ZnO/SnO_2_@NiCo_2_O_4_ preparation on carbon cloth and demonstrated a good cycling stability than NiCo_2_O_4_. In another report, J. Park et al. [2] demonstrated the utilization of NiCo_2_O_4_/PVP/PANI heterogeneous nanocomposites as an advanced battery-type electrode to achieve remarkable electrochemical supercapacitor performance. 

In previous studies, layered double hydroxides (LDHs) were widely studied as a novel transition metal-based electroactive material [52,53]. The transition metal LDH materials depend on interlayer charge reimbursing anions and positively charged brucite-like layers with the formula of [M^2+^_1−*x*_M^3+^*_x_*(OH)_2_][A*^n^*^−^]*_x_*_/n_*zH_2_O. Here, A^n−^ is the charge-reimbursing anion, and M^2+^ and M^3+^ are divalent and trivalent metal cations, respectively. Similar to a brucite-like layer, the positive charge of the layer is replaced with a trivalent metal cation in place of a divalent cation. With a large specific surface area and a layered structure, LDH material-based electrodes exhibit theoretically high specific capacitance with improved ion diffusion pathways [54]. Especially, the superior electrochemical activity of Ni-Co LDHs has been reported to enhance the capacitance and cyclic stability in charge storage systems [55,56]. Compared to mono-hydroxides like Ni(OH)_2_ and Co(OH)_2_, a synergistic effect between dual metallic components (Ni and Co) in Ni-Co LDHs was successfully obtained by providing a number of oxidation states for a faradaic redox reaction. In the successful construction of well-designed Ni-Co LDHs, morphology and structural engineering are essential strategies for energy storage devices. However, advanced architectures of high-performing materials and unique nanostructured surface designs are the keys to improving the electrochemical properties of electrodes. 

Apart from the synthesis of active materials with unique morphological features, it is important to choose a suitable substrate for active material coating, because substrates should also support the rapid transport of ions/electrons, electrochemical stability, and high durability. To date, various substrates such as nickel foam [57], graphite paper [58], copper foam [59], conductive fabric [60], carbon cloth [61], etc., are widely used in SC electrodes. However, it is difficult to modify the surface morphology of these substrates to obtain better electrochemical performance. Therefore, it is essential to explore alternative substrates that are able to change morphology with facile methodology. In addition, due to the environmental concerns associated with pollution and the demand for sustainable development, it is desirable to recycle widespread plastic waste into products of commercial value, such as high-quality carbon materials that can be used in the electrochemical field.

Considering these issues, most popular polymeric substrates like nylon, fluorinated ethylene propylene (FEP), polytetrafluoroethylene (PTFE), and polystyrene (PS), undergo glass transition under their specific operating temperature range. The glass transition of a polymer can promote variation in inherent properties such as modulus of elasticity, permittivity, the density of states, and work function. Consequently, all of these characteristics lead to significant surface modifications that affect material transfer in the surface-controlled electrode process.

Among them, PS is suggested as an advanced substrate material in the present work. As the PS is typically disposed of as solid waste after a short lifespan, the disposal of PS waste is inevitably a global issue due to its stability and non-biodegradability. Further, at the glass transition temperature, the PS substrate can inherently shrink by creating nano-to-micro surface morphology [62]. Beyond the glass transition temperature (above 150 °C), its inherent shrinkage with nano-to-micro surface roughness can be maintained even after the deposition of an electroactive layer. The electroactive material deposited onto the transitioned PS substrate not only exhibits high conductivity but also produces nano-to-micro scale crumpled morphology. Thus, without any complicated synthetic process such as etching, chemical reactions, high-level vacuum equipment, and harmful chemicals, the electroactive material can be deposited via time- and cost-effective methods with sophisticated material design. Surprisingly, based on this technique, nano-to-micro surface engineering can be easily acquired by microwave-assisted transition for only 1 min.

Based on the above description, in this work, nanostructured Ni-Co layered double hydroxides (LDHs) were hierarchically grown on micro/nanostructured conductive PS layers. With the synergistic effect of Ni and Co transition metals, the electroactive layers demonstrate their higher electronic conductivity and larger specific capacitance than those of simple transition metals. Further, tunable compositions of metal precursors (Ni and Co) enable the manipulation of the physical and chemical properties of the electroactive layer. Along with the nanostructured PS layer, optimization of Ni and Co metal ion precursors was successfully achieved to create unique micro/nanostructures by increasing the electrochemical activity. Further, based on the glass transition of PS, the as-prepared material was simply heated for only 1 min by microwave to obtain the specific nano-to-micro architecture [62]. Without using any complex equipment, the proposed hierarchical flake-like surface morphology of pseudocapacitive materials leads to significant improvement in the number of electroactive species. Consequently, this new material is beneficial for the activation of faradaic redox reactions between the surfaces of the electrode and electrolyte. Herein, the excellent performance of NiCo-LDHs/PS electrode was demonstrated, including enhanced electrochemical performance, and cycling stability. In addition, the electrochemical test results demonstrated that MWC//AC HSC device showed a high areal capacitance of 531.25 mF/cm^2^ at a current density of 5 mA/cm^2^. More importantly, the fabricated an aqueous HSC device with MWC as a positive electrode, and AC as a negative electrode showed a power density of 16 mW/cm^2^ at an energy density of 0.058 mWh/cm^2^, along with the remarkable capacity retention of 82.8% even after 10,000 continuous charging and discharging cycles. Further, the as-fabricated hybrid supercapacitor (HSC) device was successfully integrated with a triboelectric nanogenerator (TENG) for the development of energy conversion and storage systems. The surface-modified PS film also contributed to the high electric output of the TENG device (PS-TENG) by enlarging the effective contact area. Connected with the PS-TENG, the charging efficiency, as well as the feasible application of operating practical electronic devices, were studied for self-charging HSC power cells. Finally, this work not only provides a promising electrode material for improved energy conversion and storage systems but also shows the potential of an advanced structural and compositional design through simple fabrication methods.

## 2. Experimental Section

### 2.1. Materials

Nickel foam (NF), activated carbon (AC), and super P carbon were provided by MTI Korea. Cobalt nitrate hexahydrate (Co(NO_3_)_2_·6H_2_O), nickel chloride hexahydrate (NiCl_2_·6H_2_O), polyvinylidene fluoride (PVdF), and N-Methyl-2-pyrrolidone (NMP) were procured from Sigma-Aldrich. Commercial polystyrene (PS) film was purchased from MTI Korea.

### 2.2. Deposition of Thin Ni Layer onto PS Substrate

The conductive Ni layer was deposited onto the prepared PS substrate by a magnetron RF sputter at 150 W for 45 min in Ar gas at 30 sccm [62]. 

### 2.3. Preparation of Ni-Co Layered Double Hydroxides (LDHs) on Conductive PS Layer

Single and mixed metal hydroxides on PS substrate were prepared by a simple electrochemical deposition (ECD) process at room temperature (RT). Prior to the synthesis process, commercial PS substrate was deposited with conductive Ni layer by magnetron sputtering. Afterward, growth solution was prepared by adding metal precursors of NiCl_2_·6H_2_O and Co(NO_3_)_2_·6H_2_O in 30 mL of DI water. Total concentration of aqueous solution was fixed at 10 mM. During the ECD process, the PS substrate was used as working electrode along with reference (SCE) and counter (platinum) electrodes which were immersed in the above mixture solution. A chronoamperometry voltage of −1.0 V was supplied for 100 s to uniformly coat the required material on PS substrate. After the completion of coating process, all fabricated electrodes were washed and dried in a convection oven at 80 °C for 5 h. The effect of the Co-Ni ratio was extensively explored by changing the Co and Ni compositions to obtain the best electrochemical performance. Accordingly, electrodes were fabricated with different Co-Ni ratios of 1:0, 0.75:0.25, 0.5:0.5, 0.25:0.75, 0:1, which were denoted Co, Co_0.75_Ni_0.25_, Co_0.5_Ni_0.5_, Co_0.25_Ni_0.75_, and Ni, respectively. The optimized electrode is denoted Co_0.5_Ni_0.5_ throughout the manuscript.

### 2.4. Preparation of Activated Carbon (AC)-Coated Negative Electrode 

Activated carbon (AC) deposited negative electrode was prepared using the appropriate weight ratio (80:10:10) of AC, PVdF, and super P carbon. To increase the surface conductivity, a small amount of super P carbon was added to the mixture. PVdF was utilized as a binder. The slurry-type mixtures were coated and dried in a convection oven at 80 °C for 3 h.

## 3. Physical Characterization

The surface morphologies of the prepared electrodes were analyzed using field emission scanning electron microscopy (FE-SEM, Carl Zeiss, MERLIN). The elemental mapping analysis was obtained by energy-dispersive X-ray spectroscopy (EDX). The transmittance of each sample was characterized with an ultraviolet–visible spectrophotometer (UV-vis, Perkinelmer, Lambda 365). The crystallinity of the electrodes was studied by an X-ray diffractometer (XRD, Bruker, D8 Advance). The chemical stoichiometry and electronic states of the electrodes were studied by an X-ray photoemission spectroscopy (XPS, Thermo Electron Multilab 2000) with microfocus monochromated Al Kα X-rays. The porous nature, pore size distribution, and surface area were examined by nitrogen adsorption–desorption isotherm (BET) and Barrett–Joyner–Halenda (BJH) approaches in an automated gas sorption analyzer (BELSORP-max (MP)). 

### 3.1. Electrochemical Characterization

The electrochemical performance of all the electrodes and the supercapacitor device were tested and evaluated in a 1M aqueous KOH electrolyte solution under ambient conditions using an Iviumstat electrochemical instrument (IVIUM Technologies). Cyclic voltammetry (CV), galvanostatic charge–discharge (GCD), and electrochemical impedance spectroscopy (EIS) were recorded to examine the electrochemical performance of electrodes. The as-prepared free-standing electrodes were directly tested as the working electrodes. Platinum wire and Ag/AgCl were employed as the counter and reference electrodes, respectively. Further, the values of areal capacity (*Q_a__c_*) and areal capacitance (*C_a_*) were carefully calculated by the following Formulas (1) and (2) [2].

(1) $$Q_{ac}(\frac{Ah}{a})=\frac{I\times \Delta t}{a}$$

(2) $$C_{a}=\frac{I\times \Delta t}{a\times \Delta V}$$

where *I* is the applied discharge current (A), ∆*t* is the discharge time (s), *a* is the area of electroactive material (cm^2^), and ∆*V* is the potential window (V), *C*_a_ is the areal capacitance (F/cm^2^), and *Q_ac_* is the areal capacity (Ah/cm^2^).

### 3.2. Electrical Measurement

An electrodynamic shaker (Lw139.138-40, Labworks Inc., Costa Mesa, CA, USA) was utilized to create and apply vibration so as to generate periodic contact and separation between the counter triboelectric layer and dielectric material. The short-circuit current (*I*_sc_) and open-circuit voltage (*V*_oc_) produced by the fabricated device were calculated by an electrometer (Keithley 6514, Tektronix Inc., Beaverton, OR, USA).

### 3.3. Fabrication of Pouch-Type HSC Device

The hybrid supercapacitor (HSC) device, which consists of a positive electrode, negative electrode, separator, and electrolyte, was fully sealed for two electrode configurations. The suggested Ni-Co LDH coated on the PS substrate was used as a positive electrode (+ve electrode) and the activated carbon (AC) coated layer was used as a negative electrode (−ve electrode) for the assembly of HSC devices. These two electrodes were separated by filter paper and immersed in 1 M KOH electrolyte. Consequently, the areal capacitance (*C_a_*), areal energy density (*E_ad_*), and power density (*P_d_*) of the fabricated HSC device were estimated using the following Formulas (3) and (4) [2].

(3) $$E_{ad}=\frac{C_{a}\times \Delta V^{2}}{2}$$

(4) $$P_{d}=\frac{E_{ad}}{\Delta t}$$

where *E_ad_* is the areal energy density (Wh/cm^2^) and *P_d_* is the areal power density (W/cm^2^).

### 3.4. Preparation of Electrospun PVdF Membrane

In the fabrication process of the PS-TENG device, a PVdF membrane was adopted as a counter-dielectric layer by using the electrospinning method. To prepare the solution for electrospinning, 2 g of PVdF beads, 4 g of acetone, and 4 g of DMF were mixed in a beaker. Then, the mixture was stirred at 80 °C for 3 h. The solution was electrospun at 17 kV applied voltage and 3 mL/h flow rate. Finally, the membrane was dried at 80 °C for 24 h to remove residual solvent.

## 4. Results and Discussions

**Figure 1:**
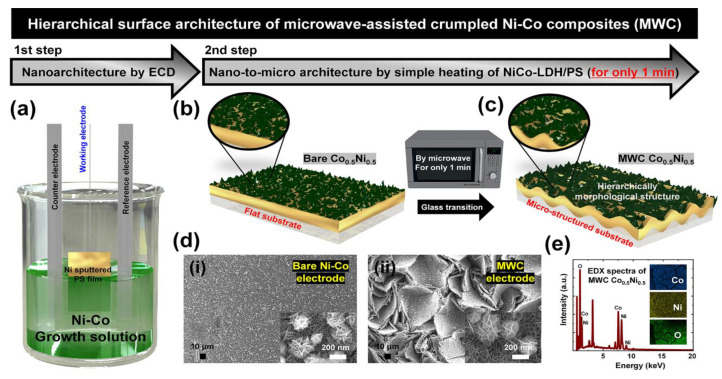
Schematic illustration of two-step synthesis of microwave-assisted crumpled Ni-Co composites (MWC) electrode with nano-to-micro structured hierarchical surface architecture (**a**) 1st step to assemble nano-architecture by electrochemical deposition (ECD) and (**b**,**c**) nano-to-micro architecture obtained by simple heating process of Ni-Co layered double hydroxides (LDHs)/PS electrode, with the assistance of microwaves for only 1 min. Material analyses including (**d**) SEM characterizations of surface morphology of bare and MWC electrodes and (**e**) EDX spectra and elemental mapping images of Co, Ni, and O in MWC Co_0.5_Ni_0.5_ electrode.

Schematic images of the fabrication method of the suggested electrode are provided in Figure 1a–c. It is well known that the glass transition temperature of the polystyrene (PS) material is 150 °C. Thus, when excess temperature over 150 °C is applied to PS, inherent surface shrinkage results in the creation of nano-to-micro scale morphology. Related to the mentioned glass transition, the prepared electrode is denoted as bare Ni-Co and microwave-assisted crumpled Ni-Co composited (MWC) electrode, respectively. Herein, the MWC is regarded as the transitioned electrode with nano-to-micro surface roughness.

In detail, through a two-step synthetic process of the electrochemical deposition (ECD) and simple heating by microwave for only 1 min, the Ni-Co composited electroactive material was successfully obtained on Ni sputtered PS substrate without any complex procedure. In the first step by ECD (Figure 1a), the Ni-Co composited nanomaterials were deposited onto a conductive PS substrate by dipping in the as-prepared growth solution. When a conductive PS substrate is immersed in a growth solution (Ni-Co compositions), the Ni and Co ions from the solution are absorbed on the surface of the working electrode (Ni-sputtered PS film). In this process, nanoarchitectures are easily achieved via the creation of the Ni-Co layered double hydroxides (LDHs) in unique surface nanomorphology. Further, the NiCo-LDHs/PS electrode resulting from the first step was heated for only 1 min to create a hierarchically designed nano-to-micro surface morphology as shown in Figure 1b,c. Compared to bare electrodes, the hierarchically morphological structure can be easily obtained in the transitioned MWC electrode by the glass transition of PS material. 

To provide a detailed study of the glass transition of the MWC electrode, the PS substrate was heated at various times (0, 10, 20, 60, and 120 s) in a microwave to optimize the transition condition for a proper heating time. As mentioned, when the obtained samples reached temperatures above the glass transition temperature of the PS material, various surface morphologies appeared with obvious shrinkage. As shown in Figure S1 (Supplementary Materials), UV–vis spectroscopy was carried out to evaluate the transparency of materials. As a result, the UV–vis transparency gradually decreased as the heating time increased, leading to specific shrinkage of as-prepared material. In detail, the transparency of transition PS materials heated for 10 and 20 s decreased slightly compared to that of bare PS. However, the transparency of transitioned PS heated for 120 s dramatically decreased because irregular light scattering occurred due to the randomly formed surface morphology. Accordingly, the optimized heating time is regarded as 60 s to obtain properly transitioned material. Thus, a further experiment was conducted in the transitioned MWC electrode based on this optimized heating condition. 

The actual surface morphology of synthesized electrodes is shown in Figure 1d. As shown in the scanning electron microscopy (SEM) results, the morphological study of bare and MWC electrodes was conducted as shown in Figure 1d(i,ii). Compared to the bare electrode (Figure 1d(i)), the MWC electrode (Figure 1d(ii)) shows randomly distributed nano-to-micro morphology. In this structure, nano-to-micro morphology from the PS substrate and nanosheets from Ni-Co LDHs created the hierarchical structure. In the inset images of Figure 1d(i,ii), the flake-like Ni-Co LDHs are well-decorated along with the bare and transitioned PS, respectively. Ultimately, without any complex fabrication process, the surface areas of synthesized electrodes can be effectively increased with the creation of unique nano-to-micro morphology. With the assistance of advanced PS substrates, Ni-Co LDHs were successfully grown as electroactive layers. For the surface elemental analysis of these, energy dispersive X-ray spectroscopy (EDX) spectra and elemental mapping images of MWC electrodes are shown in Figure 1e. The significant peaks and elemental mapping images of Ni, Co, and O confirm the presence of these elements in the synthesized MWC electrode. These elemental peaks are representative of the electroactive Ni-Co LDHs materials.

**Figure 2:**
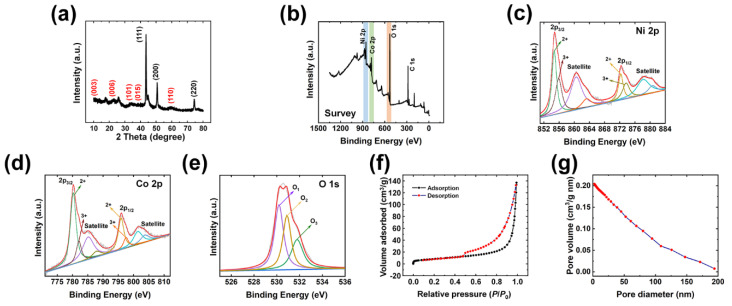
Material analysis including (**a**) X-ray diffractometer (XRD) characterization and (**b**–**e**) X-ray photoelectron spectroscopy (XPS) characterization of MWC; (**b**) survey, (**c**) Ni 2p, (**d**) Co 2p, and (**e**) O 1s spectra. (**f**) Brunauer–Emmett–Teller (BET) nitrogen adsorption-desorption isotherm and (**g**) Barrett–Joyner–Halenda (BJH) pore size distribution curve of electroactive materials of NiCo-LDHs.

As shown in Figure 2, X-ray diffraction (XRD) and X-ray photoelectron spectroscopy (XPS) characterization were conducted to further examine the MWC electrode. In Figure 2a, XRD analysis was carried out to check the phase and crystallinity. The well-defined diffraction peaks observed at 2*θ* values of 10.9°, 22.1°, 33.8°, 38.1°, and 59.2° were indexed to the (003), (006), (101), (015), and (110) planes, respectively. Rhombohedral Ni-Co LDHs were consistent with the standard XRD diffraction peaks for Ni-Co LDH (JCPDS. 33–0429) [63]. Herein, the XRD peaks at 2*θ* values of 44°, 51°, and 76° were indexed to the (111), (200), and (220) planes, respectively, with cubic structure of conductive Ni substrate.

XPS was utilized to study the chemical state, elemental vacancy, and chemical composition of the synthesized Ni-Co LDHs in MWC electrodes. Herein, the electronic states of MWC were investigated by the spectrum of survey, Ni 2p, Co 2p, and O 1s. As shown in the wide-scan survey spectrum results in Figure 2b, the peaks of Ni 2p, Co 2p, and O 1s are clearly seen, confirming the presence of each element. The C 1s signal is inevitable due to the experimental environment. For the detailed analysis of each spectrum, Figure 2c–e show the spectra of Ni 2p, Co 2p, and O 1s, respectively. These signals are responsible for the Ni-Co LDHs material in the synthesized electrode of MWC. The XPS spectrum of the Ni(2p) core levels for the Ni-Co LDHs (Figure 2c) exhibits four distinctive peaks located at the binding energy (BE) of 854.7, 860.5, 872.3, and 878.2 eV, respectively. The highly intense two peaks located at BE of 854.7 and 872.3 eV are assigned to Ni(2p) core levels, and their concomitant peaks at BE of 860.5, and 878.2 eV, respectively, are identified as respective shake-up satellite peaks. The Ni(2p) spectrum was fitted via the Voigt function following the Shirley background to precisely determine the double peak features of Ni(2p_3/2_), and Ni(2p_1/2_). The decomposed spectra corroborate perfect fit with eight peaks representing the core levels of the Ni^2+^(2p_3/2_), Ni^3+^(2p_3/2_), Ni^2+^(2p_3/2_) shakeup satellite, Ni^3+^(2p_3/2_) shakeup satellite, Ni^2+^(2p_1/2_), Ni^3+^(2p_1/2_), Ni^2+^(2p_1/2_) shakeup satellite, and Ni^3+^(2p_1/2_) shakeup satellite, respectively. The three-fold higher intensity of both the Ni(2p_3/2_) and Ni(2p_1/2_) core levels of Ni^2+^ than that of the core levels of Ni^3+^ revealed the significant appearance of Ni^2+^ cations in the NiCo-LDH material and very minor traces of Ni^3+^ formation. Likewise, to precisely determine the double peak feature of Co(2p) and its shake-up satellite peak, the Co(2p) XPS spectra were decomposed via the Voigt curve fitting function, followed by a Shirley background shown in Figure 2d. The Co(2p_3/2_) and Co(2p_1/2_) peaks were deconvoluted into four distinct peaks located at binding energies of 780.2, 785.1, 795.9, and 801.4 eV. These four peaks represent the Co^2+^(2p_3/2_), Co^3+^(2p_3/2_), Co^2+^(2p_1/2_), and Co^3+^(2p_1/2_) peaks, respectively. Moreover, the shake-up satellite peaks of Co(2p_3/2_) and Co(2p_1/2_) were also decomposed into four discrete peaks located at the binding energies of 782.1, 787.8, 797.5, and 803.6 eV. These peaks represent the shakeup satellite peaks of Co^2+^(2p_3/2_), Co^3+^(2p_3/2_), Co^2+^(2p_1/2_), and Co^3+^(2p_1/2_), respectively. The peak intensity of Co^2+^(2p_3/2_) was significantly (i.e., >2 times) larger than that of Co^3+^(2p_3/2_), indicating the presence of a substantially larger amount of Co^2+^ in the NiCo-LDH material and very minor traces of Co^3+^ formation. Figure 2e shows the deconvoluted spectrum of O 1s. The O(1s) spectrum shows three chemical states of oxygen element, respectively, labeled as O_1_, O_2_, and O_3_. The O_1_ peak at 530.2 eV corresponds to metal–oxygen bonds. The O_2_ peak at 531.0 eV is assigned to OH^−^ groups. The O_3_ peak located at 532.0 eV is identified as the physically adsorbed water. Obviously, the XPS data illustrates that the electron couples of Ni^2+^/Ni^3+^ and Co^2+^/Co^3+^ are concomitant in the as-prepared NiCo-LDH material. These XPS analyses indicate that the synthesized MWC electrode shows good crystallinity, along with phase purity.

The surface area and pore size play significant roles in determining the electrochemical performance of the fabricated electrode materials. Therefore, nitrogen adsorption–desorption isotherm studies were carried out to investigate the specific surface area and porous nature of the corresponding materials. As shown in Figure 2f, the isotherm of Ni-Co LDHs with a broad hysteresis loop in the range between 0.4 and 1 demonstrates the mesoporous nature of the material. The Brunauer–Emmett–Teller (BET) surface area of Ni-Co LDHs is observed to be 30.656 m^2^/g. The enhanced surface area can offer sufficient surface sites for the faradaic redox reaction, thereby leading to enhanced electrochemical supercapacitive performance of the electrode material. According to the Barrett–Joyner–Halenda (BJH) results shown in Figure 2g, the pore size distribution plot of Ni-Co LDHs reveals an average pore size of 3.92 nm. This pore size can efficiently favor the diffusion of electrolyte ions into the internal voids of the materials, contributing to high-rate capability. According to these results, the well-designed porous interconnection as well as the high surface area of Ni-Co LDHs was successfully confirmed for the dramatic utilization of electroactive materials. 

**Figure 3:**
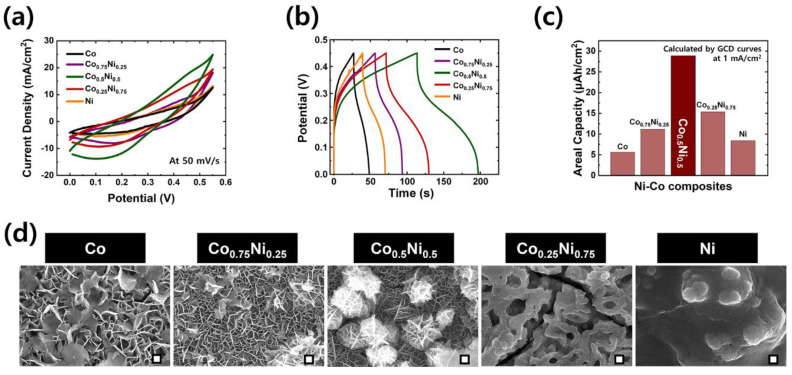
Optimization of ratios of Ni and Co in composites (Ni-Co LDHs). Comparisons of electrochemical performance for various compositions of NiCo-LDHs (Co, Co_0.75_Ni_0.25_, Co_0.5_Ni_0.5_, Co_0.25_Ni_0.75_, and Ni) through (**a**) CV (at 50 mV/s), (**b**) GCD (at 1 mA/cm^2^), and (**c**) the calculated areal capacity values from the obtained GCD results. (**d**) Comparisons of surface morphology of all compositions by FE-SEM characterization (all scale bars demonstrated in the SEM images indicate 200 nm.).

It is well known that the ratios of the metal precursors as reactants in the composition of the product can significantly affect the particle size and surface morphology. To optimize the ratios of Ni-Co composite electrodes (1:0, 0.75:0.25, 0.5:0.5, 0.25:0.75, and 0:1), the electrochemical performance of each synthesized electrode was carefully studied in a three-electrode configuration and successfully correlated with the morphological variations. Using 1.0 M KOH aqueous solution as electrolyte, cyclic voltammetry (CV) and galvanostatic charge–discharge (GCD) plots of electrodes with different Ni and Co ratios were obtained, as shown in Figure 3a,b, respectively. From these results, the optimum composite of Co_0.5_Ni_0.5_ based electrodes exhibited the highest electrochemical property. The larger integral area of the CV graph and longer charge–discharge time of the GCD graph indicate the higher capacity of the corresponding electrode. With regard to these measurements, the calculated areal capacity values are shown in Figure 3c. Areal capacity values of Co, Co_0.75_Ni_0.25_, Co_0.5_Ni_0.5_, Co_0.25_Ni_0.75_, and Ni are 5.4, 16.9, 29.3, 20.1, and 8.7 μAh/cm^2^, respectively. The abovementioned Co_0.5_Ni_0.5_ composites exhibited the best areal capacity of the five samples. This can be explained in terms of this material’s unique surface morphology, shown in Figure 3d. The specific morphological design of Ni-Co mixed hydroxide structures leads to improved electrical conductivity as well as accelerated transport of electrolyte ions during the redox reaction. Compared to the morphologies of other electrodes, such as those of Co (simple nanosheets), Co_0.75_Ni_0.25_ (nanosheets), Co_0.25_Ni_0.75_ (thick surface with a few pores), and Ni (flat film-like structures), the Co_0.5_Ni_0.5_ composites electrode exhibited the interconnected nanoflake-like morphology; some of the nanoflakes agglomerated to form spherical type structures. As expected, the controllable construction of surface morphologies promotes the synergistic effects of individual materials (Ni and Co) in the formation of interfaces between structures. Consequently, the unique surface morphology of the Co_0.5_Ni_0.5_ composites successfully resulted in faster electron transport, large electrochemically active area, decreased electron transport pathway, and short electrolyte ion diffusion length. It is revealed that the composition ratio of Co_0.5_Ni_0.5_ was the source of its superior electrochemical property. Thus, the Co_0.5_Ni_0.5_ was selected as the optimized electrode for further detailed measurements.

**Figure 4:**
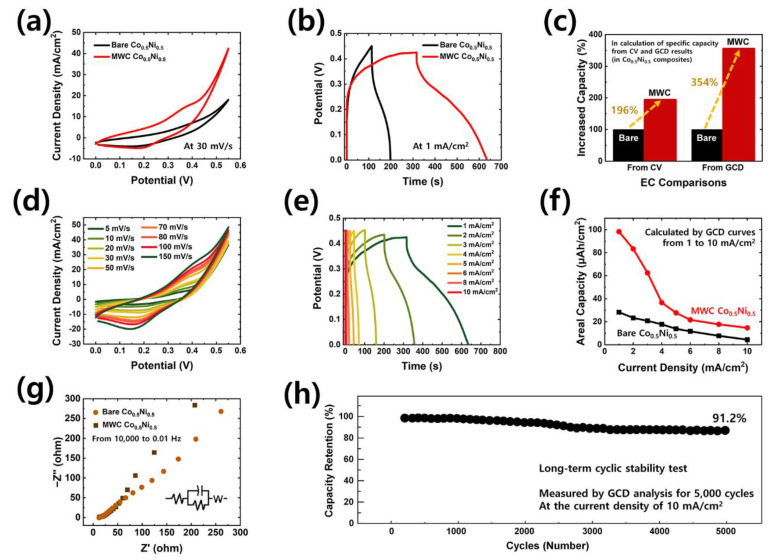
Electrochemical performance of optimized composition of Co_0.5_Ni_0.5_. Comparisons of electrochemical performance between bare and MWC through (**a**) CV (at 30 mV/s), (**b**) GCD (at 1 mA/cm^2^), and (**c**) the calculated values of increased capacity as found in CV and GCD results. Further electrochemical properties of MWC electrode with (**d**) CV (from 5 to 150 mV/s) and (**e**) GCD (from 1 to 10 mA/cm^2^). Comparisons of (**f**) areal capacity values and (**g**) EIS Nyquist plots (from 10,000 to 0.01 Hz) between bare and MWC. (**h**) Long-term cyclic stability test for 5000 GCD cycles (with capacity retention of 91.2%).

To verify the advantages of the unique morphological changes in PS substrate material at the glass transition temperature, the electrochemical properties of optimized Co_0.5_Ni_0.5_ compositions in bare and MWC electrode were compared, with results shown in Figure 4. As a result of structural and morphological variations after the heating process, the electrochemical property of MWC Co_0.5_Ni_0.5_ electrode can be seen in the CV and GCD curves to be superior to that of bare Co_0.5_Ni_0.5_ electrode. This result is confirmed by the much larger integral area of CV and longer charge–discharge rate of GCD in MWC Co_0.5_Ni_0.5_ electrode. In detail, corresponding to the redox reactions of Co^2+^/Co^3+^ and Ni^2+^/Ni^3+^, a pair of redox peaks in the CV curves can be seen in Figure 4a. Additionally, the obvious voltage drops and non-linear shapes in the GCD curves indicate the battery-type charge storage behavior of the MWC Co_0.5_Ni_0.5_ electrode. For a better comparison between bare Co_0.5_Ni_0.5_ and MWC Co_0.5_Ni_0.5_ electrodes, the exact values of increased capacity were calculated and are shown in Figure 4c. Using mathematical formulae, the areal capacity was obtained from both CV and GCD results by considering the active area. Consequently, the areal capacity of MWC was 196% (for CV) and 354% (for GCD) higher than that of bare electrode. Based on the creation of hierarchical structures, the outstandingly successful improvement of electrochemical performance in MWC electrode can be seen in these figures. As expected, the hierarchical surface morphologies lead to excellent accessibility of electrolyte ions through the electrode surface by providing larger active sites and a rapid electron transport in charge mechanisms. Finally, the outstanding performance of MWC electrode is ascribed to the remarkable advantages of its unique hierarchical structural design, originating from Ni-Co and the PS substrate. 

To examine the detailed electrochemical properties of MWC electrodes, further electrochemical measurements were conducted, and the results are shown in Figure 4d–h. In Figure 4d, the well-redox peaks observed at 0.4 V and 0.2 V indicate the faradaic reaction of Co^2+^/Co^3+^ and Ni^2+^/Ni^3+^. The reversible faraday reactions between the electrode and electrolyte interface can be described by the following redox Reactions (5) and (6) [9].

Ni(OH)_2_ + OH^−^ ↔ NiOOH + H_2_O (5)

Co(OH)_2_ + OH^−^ ↔ CoOOH + H_2_O (6)

CV curves measured at different sweep rates from 5 to 150 mV/s show similar shapes, along with a pair of strong redox peaks without any distortion noticeable in the graph. These recorded unnoticeable distortions exhibit excellent reversibility. The obvious curve as well as the obvious pair of redox peaks were also demonstrated even in the higher scan rate. These results indicate that the pseudocapacitance behavior of the charge storage mechanisms is mainly governed by faradaic redox reactions. The GCD profiles shown in Figure 4e were obtained to provide more information about the capacitive property. Under different current densities from 1 to 10 mA/cm^2^, charging and discharging graphs were drawn. As can be seen, the non-linear shape and potential drop of the GCD curves imply the battery-type characteristics of the suggested electrode. Thus, the battery-type MWC electrode successfully proves that its charge storage mechanism follows the faradaic redox reaction. Areal capacity values were carefully calculated and compared between bare and MWC electrodes as shown in Figure 4f. With different currents ranging from 1 to 10 mA/cm^2^, much higher areal capacity values of MWC electrodes were obtained, which implies this material’s superior electrochemical property. Moreover, the designed MWC Co_0.5_Ni_0.5_ electrode exhibited a superior areal capacity, which is comparable or superior to previously reported electrodes based on corresponding materials, as summarized in Table S1 (Supplementary Materials) of the Supporting Information. Due to the better charge storage capability of the MWC electrode compared to that of the bare electrode, a larger current density was obtained at the same potential bias. To investigate the charge transfer resistance and electrolyte resistance, EIS measurement (from 10,000 to 0.01 Hz) was performed, with results shown in Figure 4g. As is well known, a smaller diameter (at high frequencies) and a straight line with a higher slope (at low frequencies) may lead to better electrochemical performance. The *R*_s_/*R*_ct_ values of bare and MWC electrodes are 12.92/3.85, and 12.73/1.83, respectively. The lower *R*_s_, and *R*_ct_ values noticed in the case of the MWC electrode significantly boost the electrical conductivity and at the same time help to fasten the transfer of charges. For application to energy storage devices, this material must be subject to a long-term cycling test. Thus, the cycling stability of the MWC electrode was evaluated by GCD analysis at 10 mA/cm^2^, with results depicted in Figure 4h. Even after 5000 GCD cycles, capacitance values were approximately 91.2%, without any capacitance decay. This demonstrates the excellent electrochemical cycling stability of the electrode for long-term storage.

**Figure 5:**
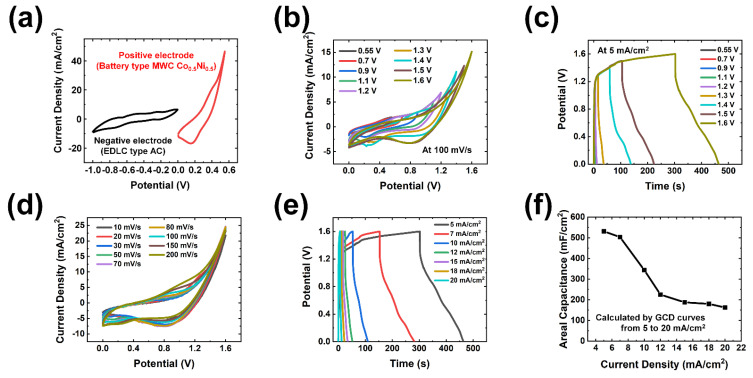
Electrochemical performance of assembled hybrid supercapacitor (HSC). (**a**) CV graphs of EDLC type AC as negative electrode (from −1.0 to 0 V) and battery type MWC Co_0.5_Ni_0.5_ as positive electrode (from 0 to 0.55 V). Electrochemical performance under potential windows from 0–0.55 to 0–1.6 V as indicated in (**b**) CV curves (at 100 mV/s) and (**c**) GCD curves (at 5 mA/cm^2^). Detailed measurement under extended potential window from 0 to 1.6 V in (**d**) CV graphs (from 10 to 200 mV/s) and (**e**) GCD graphs (from 5 to 20 mA/ cm^2^). (**f**) Areal capacitance values calculated by GCD.

In the aforementioned studies, the superior electrochemical performance of synthesized positive electrodes (MWC) was successfully demonstrated via various characterizations. Further, to completely test the energy storage performance of the final device for practical application, the suggested battery type positive electrode was assembled with EDLC type negative electrode. The preparation and electrochemical analysis of the negative electrode (i.e., activated carbon (AC)) is specified in Figure S2 (Supplementary Materials). In the fabricated hybrid supercapacitor (HSC), a different charge storage mechanism between positive and negative electrodes can be facilitated to achieve higher energy and power density, respectively. With the synergistic effect of capacitive and Faradaic reactions in the HSC device, charges from the rapid charge transfer (between the MWC electrode and KOH aqueous electrolyte) can easily accumulate on the surface of the porous structured AC. In Figure 5a, it can be seen that the operating voltage of the negative electrode (EDLC type AC) was from −1.0 to 0 V and that of the positive electrode (battery type MWC) was from 0 to 0.55 V. By integrating these positive and negative electrodes to construct an energy storage system, an operating voltage of 1.6 V was obtained in the assembled HSC device.

Figure 5b(i) shows the electrochemical performance of a fabricated HSC device measured in a two-electrode system. CV and GCD curves obtained in the extended voltage window of 0–1.6 V are shown in Figure 5b,c, respectively. Over various voltage windows, no obvious distortions were found in the graphs. The quasi-rectangular shaped CV curves (at 100 mV/s) without obvious redox peaks suggest a remarkable rate capability and higher capacitance; the GCD curves (at 5 mA/cm^2^) showed a clear *IR* drop of 0–1.6 V. These results originated from the characteristics of an ideal HSC device, which is a combination of EDLC and battery type electrodes. Accordingly, the maximum voltage window of the assembled MWC//AC HSC was successfully extended to 0–1.6 V, and further measurement was conducted within this potential range.

CV curves recorded at various scan rates (from 10 to 200 mV/s) and GCD cycle results obtained at various current densities (from 5 to 20 mA/cm^2^), shown in Figure 5d,e, respectively, show the detailed electrochemical performance. In Figure 5d, the quasi-rectangular shapes of CV were maintained without drastic change, and with no redox peaks. Similar behavior of the discharging curves was noticed, suggesting the excellent rate performance capability of MWC//AC HSC (Figure 5e). The above measurements demonstrate the promising electrode characteristics of MWC for HSCs. To further determine the charge storage performance, the areal capacitance values were carefully calculated according to the obtained GCD curves, shown in Figure 5e. The areal capacitance values decreased linearly with increases in current density (Figure 5f). As a result, the MWC//AC HSC device showed a high areal capacitance of 531.25 mF/cm^2^ at a current density of 5 mA/cm^2^. The specific capacitances at different current densities of 5, 7, 10, 12, 15, 18, and 20 mA/cm^2^ were 531.25, 503.12, 343.75, 225, 187.5, 180, and 162.5 mF/cm^2^, respectively.

**Figure 6:**
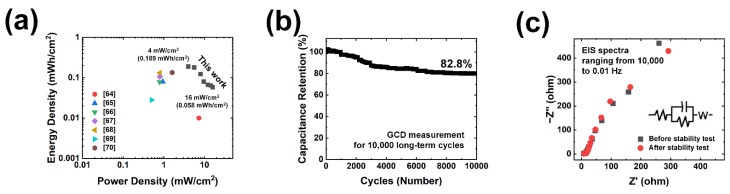
Electrochemical performance of assembled hybrid supercapacitor (HSC). (**a**) Ragone plots of energy and power densities of fabricated HSC device. (**b**) Long-term cyclic stability test for 10,000 GCD cycles (with high capacitance retention of 82.8%) and (**c**) EIS Nyquist plots (obtained from 10,000 to 0.01 Hz) before and after cyclic test.

Further, the power density and energy density of the suggested MWC//AC HSC are essential for real-time applications. The Ragone plot in Figure 6a shows calculated power density and energy density, illustrating the *E–P* relationship. From these results, a power density of 16 mW/cm^2^ at an energy density of 0.058 mWh/cm^2^ and a power density of 4 mW/cm^2^ at an energy density of 0.189 mWh/cm^2^ was obtained. The device delivers maximum energy density and power density values that are better than the many previously reported devices as shown in Table S2 (Supplementary Materials) [64,65,66,67,68,69,70]. Finally, cycling stability was considered a crucial parameter for evaluating the performance of HSC devices. As can be seen in Figure 6b, an ultra-high capacitance retention value of 82.8% was achieved even after the long-term GCD cycling test (10,000 GCD cycles). Surprisingly, there is no dramatic change in shape of the EIS curve after the stability test, as shown in Figure 6c. The *R*_s_ values were 7.63 and 7.37 Ω, equivalent to the device before and after the cyclic stability test, respectively, while the *R*_ct_ values were 0.67 and 0.14 Ω, equivalent to the device before and after the cyclic stability test, respectively. With the minor variations in *R*_s_ and *R*_ct_, the device revealed good conductivity even after 10,000 continuous charging and discharging cycles. Ultimately, all these studies reveal the superior electrochemical properties of the fabricated devices over a long period of continuous utilization in power electronic devices.

**Figure 7:**
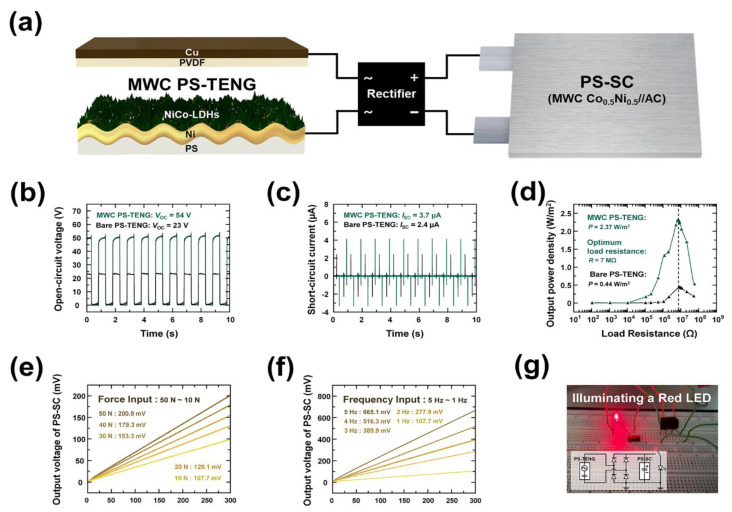
Triboelectric-driven self-charging HSC power cells composed of as-fabricated MWC PS-TENG and PS-SC. (**a**) Schematic circuit diagram of integrated energy system. Electrical output performance for comparison of bare PS-TENG and MWC PS-TENG; (**b**) open-circuit voltage (*V*_oc_), (**c**) short-circuit current (*I*_sc_), and (**d**) output power density for the dependence of the load resistance. The charging efficiency under (**e**) various input forces (from 10 to 50 N), and (**f**) input frequencies (from 1 to 5 Hz) of MWC PS-TENG. (**g**) Demonstration of feasible application by lighting up a commercial LED (in inset image of circuit diagram).

Based on the advanced electrochemical performances of as-fabricated HSC devices (MWC//AC), a self-powered energy conversion and storage system was developed to confirm the properties of an integrated system (in Figure 7). For the construction of such an integrated system, different approaches using energy harvesters (by PS-triboelectric nanogenerator (TENG)) and energy storage systems (by PS-SC) were combined using a rectifier. According to the demonstrated circuit diagram of the triboelectric-driven self-charging HSC power cells (shown in Figure 7a), the alternating output current was rectified to direct current to store electrical energy in the HSC device. Here, the periodical contact separation mode of the PS-TENG was used as an input power source to generate electrical energy via a combination of triboelectrification (also known as contact electrification) and electrostatic effect. For a simple PS-TENG model, the electrospun PVdF membrane acted as a counter triboelectric layer with the suggested MWC material. As was noted earlier, the wrinkled PS-based electrode results in a highly improved surface area, which enables an enlarged effective contact area. According to the improved surface area, as shown in Figure 7b–d, the much-enhanced electrical output performance of the MWC electrode is confirmed via comparison with bare PS-TENG. The MWC PS-TENG showed 54 V of open-circuit voltage (*V*_oc_) and 3.7 µA of short-circuit current (*I*_sc_), while the bare PS-TENG. showed 23 V of *V*_oc_ and 2.4 µA of *I*_sc_. These results imply that the improved electrical output performance of the MWC PS-TENG resulted from the very large surface charge density due to the wrinkled structure. Based on these results, the maximum power was characterized by various load resistors in a range of 100 Ω to 50 MΩ. Comparing bare PS-TENG and MWC PS-TENG, the maximum output power was usually found at moderate resistance levels. As shown in Figure 7d, the maximum instantaneous output power density of MWC PS-TENG was 2.37 W/m^2^, with a load resistance of approximately 7 MΩ. In contrast, bare PS-TENG exhibited a value of only 0.44 W/m^2^ at a similar load resistance value.

Considering the superior performance of MWC PS-TENG, device capacity for self-powered energy conversion and storage was confirmed to be due to the mechanism of the HSC, which is charged with energy generated by the MWC PS-TENG. The self-charging profiles of the integrated system under various input forces and input frequencies are depicted in Figure 7e,f, respectively. In Figure 7e, the effect of varying the magnitude of the applied force on the charging efficiency is shown. The rate of charging was gradual during the increase in input forces. As a result, charging values of 107.7, 129.1, 153.3, 179.3, and 200.9 mV were successfully obtained with input forces ranging from 10 to 50 N. In addition to this, the effects of different frequencies were evaluated, with the results shown in Figure 7f. The charging rate gradually increased with the increase in input frequency. In detail, charging values 107.7, 277.9, 389.9, 516.3, and 665.1 mV were successfully obtained with input frequencies in a range of 1 to 5 Hz. Finally, the device can be charged to about 0.7 V within only 300 s via periodical contact separation under 10 N of input force and 5 Hz of operating frequency.

On the basis of the outstanding charging efficiency of the suggested integrated system, a feasible device application was proven by lighting up several commercial LEDs. With storage of the electrical energy generated by MWC PS-TENG, a red LED was brightly lit up, proving that this mechanism can instantly power up small devices (as shown in Figure 7g). The circuit diagram of the designed self-powered energy storage system is provided in the inset image of Figure 7g. Based on the above features, the viability of the self-powered operation of the integrated device, as well as the outstanding charging efficiency, was successfully proven for practical applications such as sustainable wearable electronics and smart sensing systems.

## 5. Conclusions

In summary, the electroactive Ni-Co LDHs nanosheets were easily grown onto Ni sputtered polystyrene (PS) substrate by electrochemical deposition (ECD) method. The composites with different ratios of Co-Ni precursors were synthesized by varying the mole ratio of Co and Ni. XRD and XPS studies revealed the perfect phase purity of the prepared samples, which showed no presence of impurities. The MWC Co_0.5_Ni_0.5_ electrode was optimized with a well-designed layered-double hydroxide (LDH) structure and exhibited outstanding electrochemical performance. In particular, the synthesized LDH morphology enhances the electronic structure as well as the electric conductivity, offering a huge number of active sites and a rapid electron transfer process. Furthermore, battery-type MWC Co_0.5_Ni_0.5_ as a positive electrode and a capacitive-type activated carbon (AC) layer as a negative electrode were combined to fabricate a hybrid supercapacitor (HSC) device. The as-fabricated HSC exhibits superior energy storage performance with a high areal capacitance value of 531.25 mF/cm^2^ at a current density of 5 mA/cm^2^. Moreover, the HSC device retains 82.8% of its initial areal capacitance after 10,000 cycles of GCD measurements. On the basis of the above-mentioned energy storage performance, a triboelectrification-empowered self-charging power cell was successfully developed to instantly scavenge ambient mechanical energy and thereby store it for sustainable power generation. Therefore, this work illustrates that the MWC electrode has excellent potential for energy conversion and storage systems.

## Data Availability

The data presented in this study are available on request from the corresponding author.

## Supplementary Materials

The following are available online at https://www.mdpi.com/article/10.3390/polym15020454/s1, Figure S1. UV-vis spectroscopy results for characterization of bare and transitioned PS material (heated for 10, 20, 60, and 120 sec). Table S1. Comparative areal capacity of MWC Co_0.5_Ni_0.5_ electrode with the previously reported mono/core-shell-like transition metal hydroxide/oxide/chalcogenide-based electrodes. Figure S2. The electrochemical performance of the negative electrode by (a) CV (from 5 to 50 mV/s of scan rates) and (b) GCD analysis (from 5 to 12 mA/cm2 of current densities). Table S2. Comparison of energy density and power density of MWC//AC HSC with other previously-reported supercapacitor devices [66,71,72,73,74,75,76,77,78,79,80].
