# Supplementary material for: Microwave-Assisted Hierarchically Grown Flake-like NiCo Layered Double Hydroxide Nanosheets on Transitioned Polystyrene towards Triboelectricity-Driven Self-Charging Hybrid Supercapacitors
Source: Polymers (Basel). 2023 Jan 15;15(2):454. doi: 10.3390/polym15020454 (PMC9864052; doi:10.3390/polym15020454)
Supplement: Supplementary file 1 [file polymers-15-00454-s001.zip › polymers-2135549-supplementary.pdf]

polymers

Supplementary Materials

Microwave-Assisted Hierarchically Grown Flake-Like NiCo Layered Double Hydroxide Nanosheets on Transitioned Polystyrene towards Triboelectricity-Driven Self-Charging Hybrid Supercapacitors

Seungju Jo [1,3], Narasimharao Kitchamsetti [2,3], Hyunwoo Cho [1,3] and Daewon Kim [2,3,*]

[1] Department of Electronics and Information Convergence Engineering, Kyung Hee University, 1732 Deogyeong-daero, Giheung-gu, Yongin 17104, Republic of Korea
[2] Department of Electronic Engineering, Kyung Hee University, 1732 Deogyeong-daero, Giheung-gu, Yongin 17104, Republic of Korea
[3] Institute for Wearable Convergence Electronics, Kyung Hee University, 1732 Deogyeong-daero, Giheung-gu, Yongin 17104, Republic of Korea
* Correspondence: daewon@khu.ac.kr

Citation: Jo, S.; Kitchamsetti, N.; Cho, H.; Kim, D. Microwave-Assisted Hierarchically Grown Flake-Like NiCo Layered Double Hydroxide Nanosheets on Transitioned Polystyrene towards Triboelectricity-Driven Self-Charging Hybrid. Supercapacitors. *Polymers* **2023**, *15*, x. https://doi.org/10.3390/xxxxx

Academic Editors: Mohamed Bassyouni, Mohamed Helmy Abdel-Aziz, Shereen M. S. Abdel-Hamid, Mohamed Shafick Zoromba and Yasser Elhenawy

Received: 17 December 2022
Revised: 8 January 2023
Accepted: 11 January 2023
Published: date

Copyright: © 2023 by the authors. Licensee MDPI, Basel, Switzerland. This article is an open access article distributed under the terms and conditions of the Creative Commons Attribution (CC BY) license (https://creativecommons.org/licenses/by/4.0/).

Table of contents

1. Characterization of PS materials by UV-vis spectroscopy
2. Table S1. Comparative areal capacity of MWC $Co_{0.5}Ni_{0.5}$ electrode with the previously reported mono/core-shell-like transition metal hydroxide/oxide/chalcogenide-based electrodes
3. The electrochemical performance of the negative electrode
4. Table S2. Comparison of energy density and power density of MWC//AC HSC with other previously reported supercapacitor devices

1. Characterization of PS materials by UV-vis spectroscopy

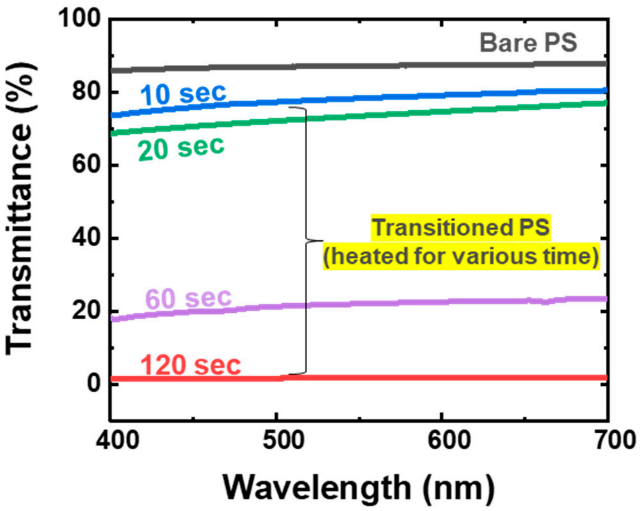

Figure S1. UV-vis spectroscopy results for characterization of bare and transitioned PS material (heated for 10, 20, 60, and 120 sec).

2. Table S1. Comparative areal capacity of MWC $Co_{0.5}Ni_{0.5}$ electrode with the previously reported mono/core-shell-like transition metal hydroxide/oxide/chalcogenide-based electrodes.

Samples	Electrolyte	Test condition (mA/cm^2)	Areal capacity (μAh/cm^2)	Ref.
$NiCo_2Al$ LDH/CC	1 M KOH	0.8	237	S1
$Ag@Ce_6Mo_{10}O_{39}$	1 M KOH	2	62	S2
NiCo LDH@Ag/CC	1 M KOH	1	142	S3
Ni-Co@Ni-Co LDH NTAs	1 M NaOH	4.5	275	S4
$CoMoO_4@Co(OH)_2$	2 M KOH	1	265	S5
$Co@Co(OH)_2$/ Cellulose paper	6 M KOH	2	89	S6
$Ni_3S_4@Co_9S_8$	2 M NaOH	2.4	367.47	S7
NiO-Co_3O_4-NiO/NF	1 M KOH	4	314	S8
$Cu_{1-x}Ni_x$-O/Ni– P/Cardboard paper	1 M KOH	2	331	S9
$CuCo_2S_4/CuCo_2O_4$	2 M KOH	1	~180.5	S10
$Ni_{1.5}Co_{1.5}S_4@Ti_3C_2$	2 M KOH	2	~333.4	S11
**MWC $Co_{0.5}Ni_{0.5}$**	**1 M KOH**	**1**	**100**	**This work**

3. The electrochemical performance of the negative electrode

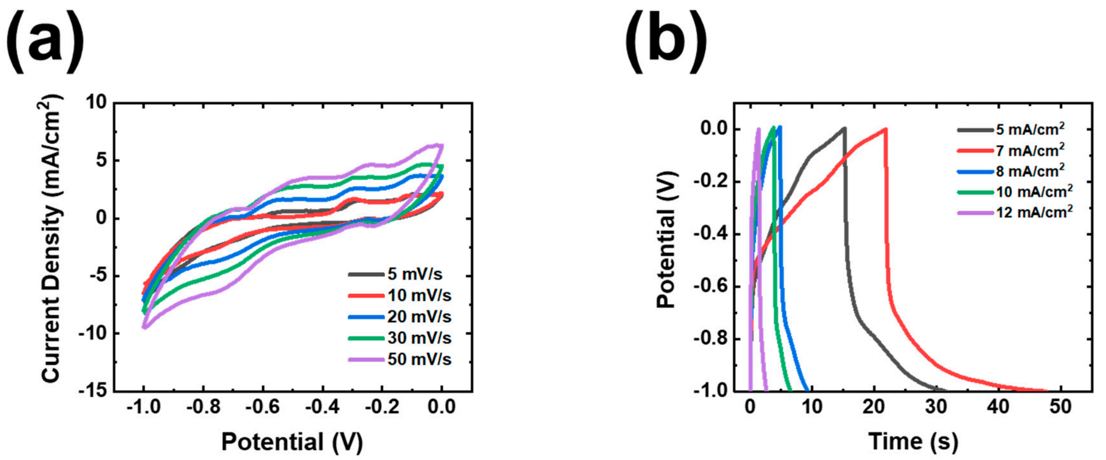

Figure S2. The electrochemical performance of the negative electrode by (**a**) CV (from 5 to 50 mV/s of scan rates) and (**b**) GCD analysis (from 5 to 12 mA/cm^2 of current densities).

4. Table S2. Comparison of energy density and power density of MWC//AC HSC with other previously-reported supercapacitor devices

Supercapacitor device	Electrolyte	Potential window (V)	Energy density (mWh/cm^2)	Power density (mW/cm^2)	Ref.
$Ni(OH)_2$ NWs//Carbon fiber	PVA/KOH	1.5	0.01	7.3	[64]
$Ni(OH)_2$-NG//NG	PVA/H_2SO_4	1.45	0.0795	0.944	[65]
Ag@NiCo-LDH//AC	KOH	1.6	0.0788	0.785	[66]
CoN-Ni_3N/N-C//VN	PVA/LiOH	1.6	0.106	0.8	[67]
Co_9S_8@PPy@NiCo-LDH NTAs//AC	PAAm/LiOH	1.6	0.132	0.8	[68]
Co_3O_4/C	PVA/KOH	1	0.028	0.5	[69]
NiCo-LDH@$Ti_3C_2T_x$//AC	PVA/KOH	1.6	0.134	1.61	[70]
**This work**	KOH	1.6	0.189	4	-

References

1. X. Gao, X. Liu, D. Wu, B. Qian, Z. Kou, Z. Pan, Y. Pang, L. Miao, J. Wang, Significant role of al in ternary layered double hydroxides for enhancing electrochemical performance of flexible asymmetric supercapacitor, Adv. Funct. Mater. 29 (36) (2019) 1903879.
2. S.C. Sekhar, G. Nagaraju, B. Ramulu, J.S. Yu, Hierarchically designed $Ag@Ce_6Mo_{10}O_{39}$ marigold flower-like architectures: an efficient electrode material for hybrid supercapacitors, ACS Appl. Mater. Interfaces. 10 (43) (2018) 36976-36987.
3. S.C. Sekhar, G. Nagaraju, J.S. Yu, Conductive silver nanowires-fenced carbon cloth fibers-supported layered double hydroxide nanosheets as a flexible and binder-free electrode for high-performance asymmetric supercapacitors, Nano Energy 36 (2017) 58-67.
4. Y. Liu, N. Fu, G. Zhang, M. Xu, W. Lu, L. Zhou, H. Huang, Design of hierarchical NiCo@NiCo layered double hydroxide core–shell structured nanotube array for high-performance flexible all-solid-state battery-type supercapacitors, Adv. Funct. Mater. 27 (8) (2017) 1605307.
5. G.K. Veerasubramani, A. Chandrasekhar, S. M. S. P, Y.S. Mok, S.J. Kim, Liquid electrolyte mediated flexible pouch-type hybrid supercapacitor based on binder less core–shell nanostructures assembled with honeycomb-like porous carbon, J. Mater. Chem. A. 5 (22) (2017) 11100-11113.
6. C. Wan, Y. Jiao, D. Liang, Y. Wu, J. Li, A Geologic Architecture System-Inspired Micro-/Nano-Heterostructure Design for High-Performance Energy Storage, Adv. Energy Mater.8 (33) (2018) 1802388.
7. H. Li, F. Yue, H. Xie, C. Yang, Y. Zhang, L. Zhang, J. Wang, Hollow shell-in-shell $Ni_3S_4@Co_9S_8$ tubes derived from core–shell Ni-MOF-74@Co-MOF-74 as efficient faradaic electrodes, CrystEngComm. 20 (7) (2018) 889-895.
8. S. Chandra Sekhar, G. Nagaraju, J.S. Yu, High-performance pouch-type hybrid supercapacitor based on hierarchical NiO-Co_3O_4-NiO composite nanoarchitectures as an advanced electrode material, Nano Energy 48 (2018) 81-92.
9. G. Nagaraju, S.C. Sekhar, B. Ramulu, G.K. Veerasubramani, D. Narsimulu, S.K. Hussain, J.S. Yu, An agriculture-inspired nanostrategy towards flexible and highly efficient hybrid supercapacitors using ubiquitous substrates, Nano Energy 66 (2019) 104054.
10. X. Xu, Y. Liu, P. Dong, P.M. Ajayan, J. Shen, M. Ye, Mesostructured $CuCo_2S_4/CuCo_2O_4$ nanoflowers as advanced electrodes for asymmetric supercapacitors, J. Power Sources. 400 (2018) 96-103.
11. X. He, T. Bi, X. Zheng, W. Zhu, J. Jiang, Nickel cobalt sulfide nanoparticles grown on titanium carbide MXenes for high-performance supercapacitor, Electrochim. Acta 332 (2020) 135514.
